# Adult congenital pulmonary valve insertion using a new bioprosthetic aortic valve: Inspiris

**DOI:** 10.1002/ccr3.2794

**Published:** 2020-03-31

**Authors:** Chi Chi Do‐Nguyen, Maxwell F. Kilcoyne, Randy M. Stevens, James Starc, Nandini Madan, Vicki Mahan, Cesar Igor Mesia, Achintya Moulick

**Affiliations:** ^1^ Philadelphia College of Osteopathic Medicine Philadelphia Pennsylvania; ^2^ Department of Cardiothoracic Surgery St. Christopher’s Hospital for Children Philadelphia Pennsylvania

**Keywords:** cardiothoracic surgery, cardiovascular disorders

## Abstract

We describe successful placement of the Inspiris Resilia aortic valve in the pulmonary position. This valve has advantages for immediate benefit and future percutaneous interventions, making it a promising prosthesis for adult congenital patients.

## Graphical abstract

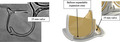

## 1 INTRODUCTION

A 20‐year‐old man born with pulmonary atresia with intact ventricular septum status postneonatal radiofrequency pulmonary valvotomy presents with long‐standing right ventricular enlargement and pulmonary insufficiency. He was not a candidate for transcatheter pulmonary valve placement and underwent surgical placement of a 25 mm Inspiris Resilia valve.

Pulmonary atresia with an intact ventricular septum (PA‐IVS) is characterized by a complete anatomical obstruction of blood flow from the right ventricle (RV) to the pulmonary trunk by an imperforate pulmonary valve (PV).^1^ Transcatheter pulmonary valvotomy (TPV) can palliate selected patients in the neonatal period, but patients are left with lifelong pulmonary insufficiency (PI).^2^, ^3^ Currently, no surgical valve prosthesis is designed for the PV and thus, patients requiring PV replacement (PVR) receive aortic valve prosthesis in the pulmonary position. This is the first report of surgical PVR (SPVR) using the Inspiris Resilia aortic valve in a patient with PI after neonatal TPV for PA‐IVS.

## 2 CASE REPORT

A 20‐year‐old male with a history of PA‐IVS, which was managed with TPV and balloon angioplasty as a neonate, originally presented for routine follow‐up at 15 years old. During this time, the patient was asymptomatic and underwent a cardiac MRI revealed a RV end‐diastolic volume (RVEDVI) of 154 mL/m^2^, indicating severe RV volume overload, and suggested that he was a possible candidate for implantation of a transcatheter pulmonary valve (TPVR).

**Figure 1:**
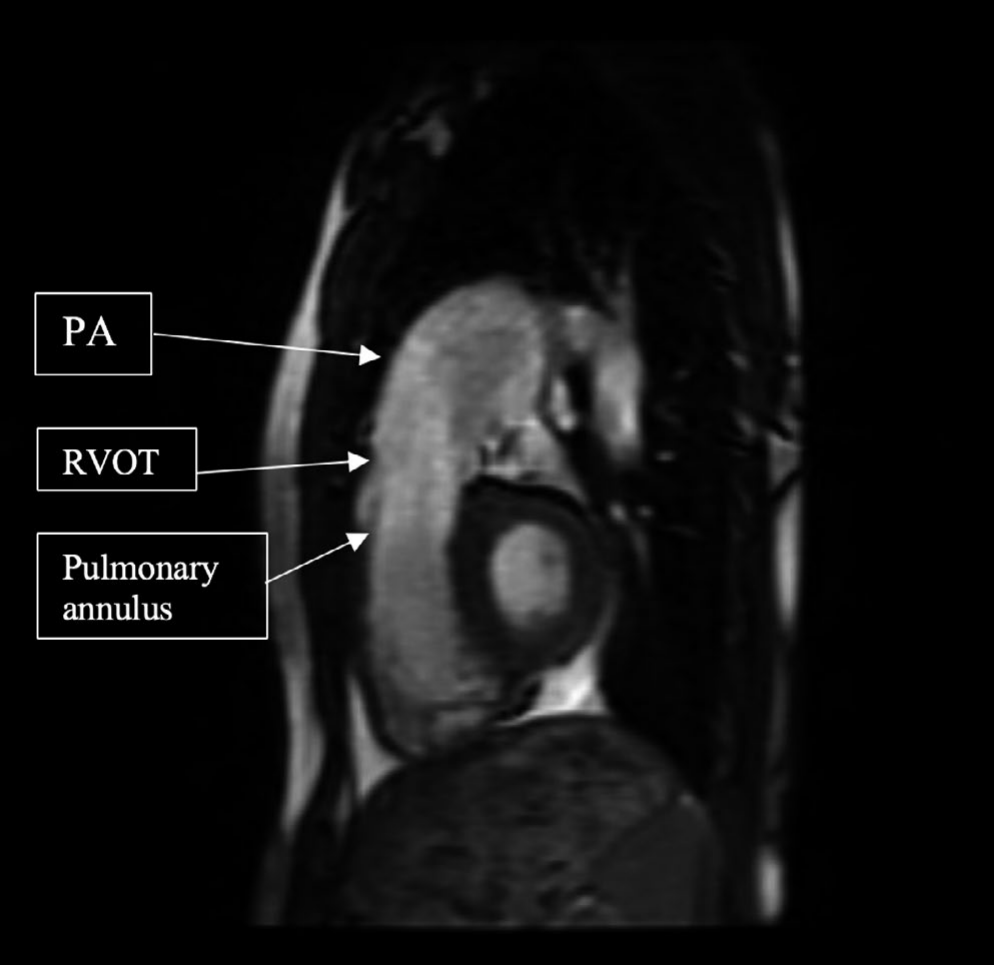
Sagittal right ventricular outflow tract (RVOT) view on cardiac MRI demonstrating the enlarged PA (43 × 41 mm), RVOT (27 × 35 mm) and pulmonary annulus (38 × 36 mm)

**Figure 2:**
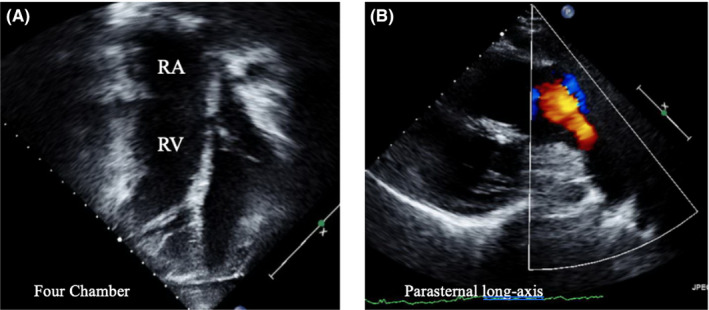
Preoperative four‐chamber (A) and parasternal long‐axis (B) views illustrating a dilated RV and the broad red jet of PI, respectively

During the workup for TPVR, he underwent cardiac catheterization at 17 years old which confirmed the extent of RV enlargement, but also implied that the main pulmonary artery (PA) was too large for TPVR. The patient declined SPVR and remained in follow‐up. At 20 years old, he had developed progressive ventricular ectopy and a repeat MRI confirmed the initial findings of severe RV and PA (43 × 41 mm) dilation (Figure 1). The RVEDVI and RV end‐systolic volume (RVESVI) were 178.9 mL/m^2^ and 80.3 mL/m^2^, respectively. An echocardiogram revealed a moderately dilated RV with preserved systolic function (55%), normal left ventricular function (65%), and severe PI (Figure 2). Per the 2018 American Heart Association/American College of Cardiology Guidelines (Table 1), he met the indications for PVR and agreed to proceed with SPVR.^4^

**Table 1:** Criteria for Isolated PVR for Asymptomatic PR

Moderate or severe PI and RV enlargement
And two of the following:
Mild or moderate RV or LV systolic dysfunction Severe RV dilation (RVEDVI > 160 mL/m^2^, RVESVI >80 mL/m^2^, or RVEDV >2× LVEDV) RVSP due to RVOT obstruction >2/3 systemic pressure Progressive reduction in objective exercise tolerance

**Figure 3:**
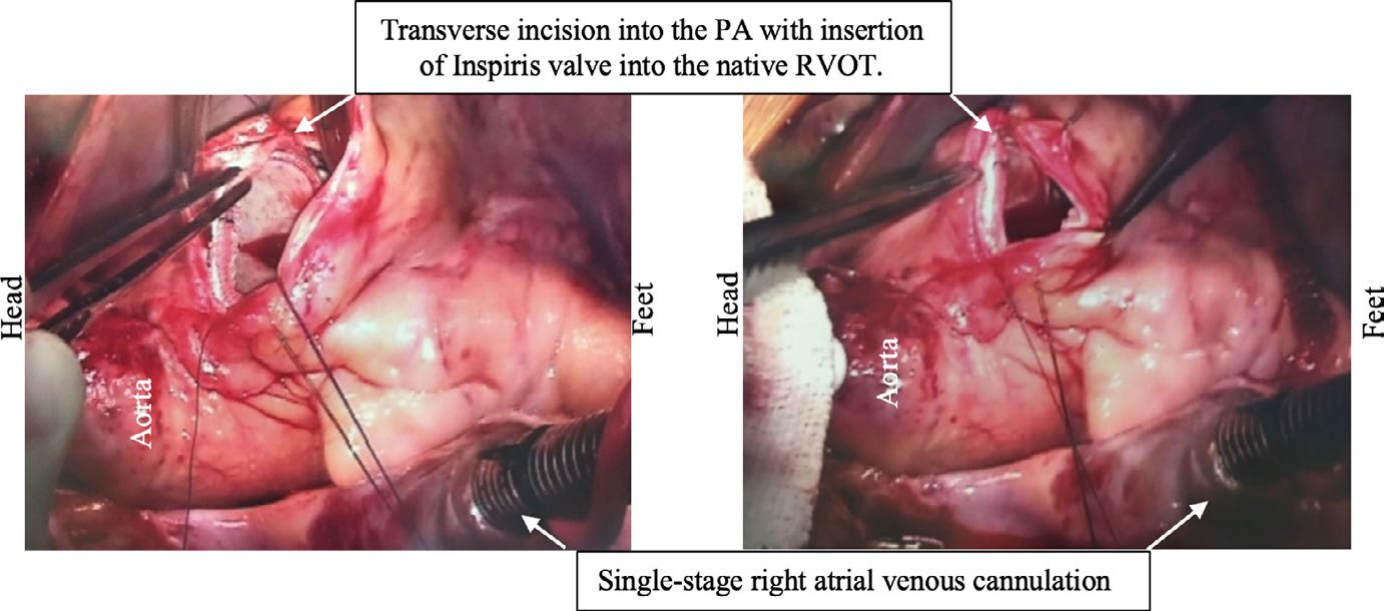
Intraoperative images of Inspiris Resilia valve insertion with continuous simple running suture to the native RVOT and primary closure of the PA with no patch required

**Figure 4:**
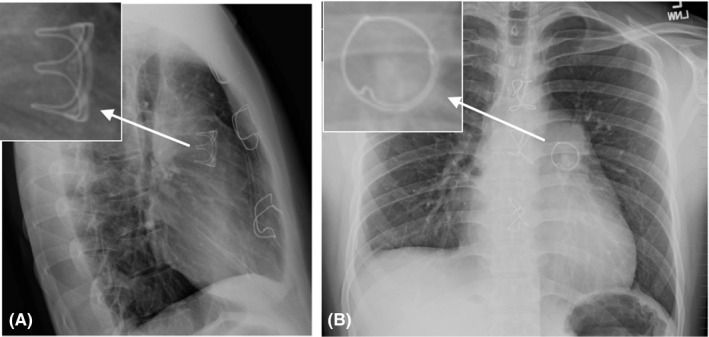
Postoperative chest radiograph in lateral (A) and PA view (B), showing the 25 mm Inspiris valve in place

**Figure 5:**
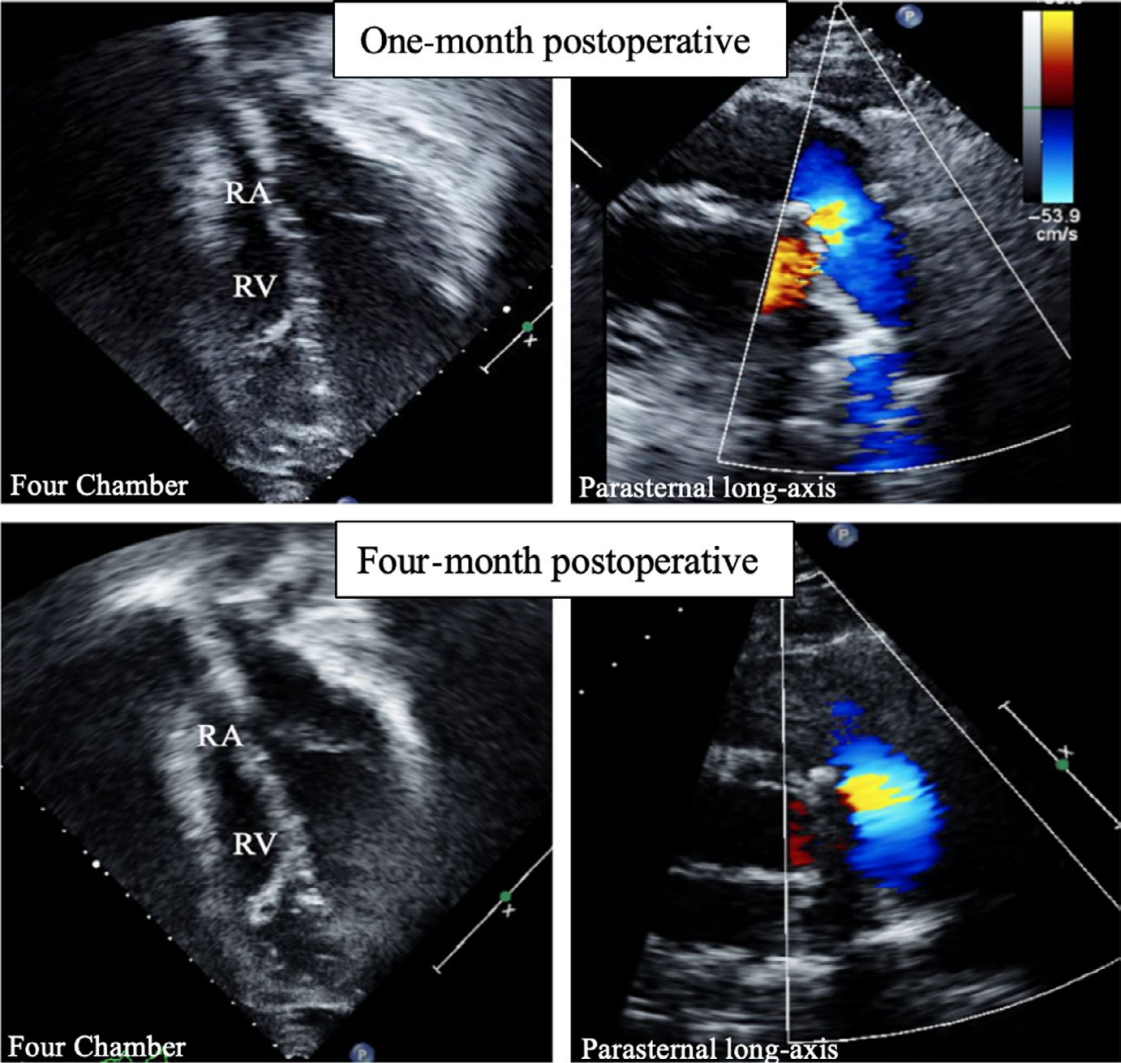
One‐ and four‐month postoperative four‐chamber and parasternal long‐axis views demonstrating normalization of RV volume and no PI, respectively

He was taken to the operating room and underwent an on‐pump beating heart pulmonary valve insertion using the 25 mm Inspiris Resilia bioprosthetic valve. The valve was inserted into the pulmonary position with continuous prolene suture to the native right ventricular outflow tract (RVOT). The PA did not require a patch for closure (Figures 3 and 4). He was discharged home on postoperative day #5 on oral diuretics. The patient was readmitted to the ICU on postoperative day #19 due to worsening bilateral pleural effusions and underwent thoracentesis and an 8.5 French pigtail catheter placement before being discharged from the hospital on postoperative day #21 on increased oral diuretics. His echocardiogram at 1 (Figure 5) and 4 months postoperatively demonstrated no significant gradient across the PV, no paravalvular leak, normalization of RV pressures, and no PI.

## 3 DISCUSSION

In selected patients, PA‐IVS can be palliated with TPV and balloon dilation in the neonatal period. This approach offers lower morbidity and mortality compared with upfront surgery but leads to progressive PI in 44%‐88% of patients.^3^, ^5^ The adult congenital management of these patients remains SPVR, but the prosthesis to use at the pulmonary position continues to be a topic of debate. The Inspiris Resilia valve is a bovine pericardial bioprosthetic valve and our use of this valve was based on its hemodynamics, durability, and unique valve‐in‐valve features, making it amenable to the adult congenital population (Table 2).

**Table 2:** Potential Benefits of Inspiris Resilia Valve

Size markers that are fluoroscopically visible allowing for lifelong size identification Expansion zone that allows for several millimeters of expansion during subsequent transcatheter valve replacement Anticalcium properties and glycerol coating aimed to enhance durability

In the aortic position, the Inspiris Resilia valve has been demonstrated to have good hemodynamics, including lower mean pressure gradients (13.9 ± 6.1 mm Hg) and a large effective orifice area (EOA) (1.8 ± 0.6 cm^2^), compared with Carpentier‐Edwards, Mosaic, and Epic valves at 1‐year.^6^ Studies with up to 1‐year of follow‐up showed there was no significant change in EOA and mean pressure gradients, and a paravalvular leak rate of <1%.^6^ In addition, adult congenital cardiac patients may have a small annulus and are at high risk for patient prosthetic mismatch. For the 19 mm prosthesis, the Inspiris Resilia valve demonstrated a mean EOA index of 0.8 ± 0.2 at 3‐6 months follow‐up which is superior to the Medtronic Mosaic Ultra valve (0.66 ± 0.15) and comparable to the Carpentier‐Edwards Perimount Magna valve (0.93 ± 0.21).^6^, ^7^

The Inspiris Resilia valve has features aimed to enhance its durability, which is especially important in younger patients who have an increased propensity for valve degeneration and rapidly progressive valve stenosis.^8^ The Inspiris Resilia valve has unique anticalcium binding properties and a glycerol coating which helps preserve the integrity of the valve by displacing fluid in pericardial tissues.^6^, ^9^ While clinical studies have yet to demonstrate long‐term durability of the Inspiris Resilia valve in humans, preclinical in vivo studies have been promising. In a randomized controlled trial in juvenile sheep, the Inspiris Resilia valve had significantly less calcium degeneration compared with the PERIMOUNT bioprosthesis using radiographic and histologic analysis.^10^ In another animal model, tissue disks, comprised of either Inspiris Resilia or XenologicX tissue, were injected intramuscularly in rabbits and also found significantly less calcific degeneration in the Inspiris Resilia tissue.^11^

**Figure 6:**
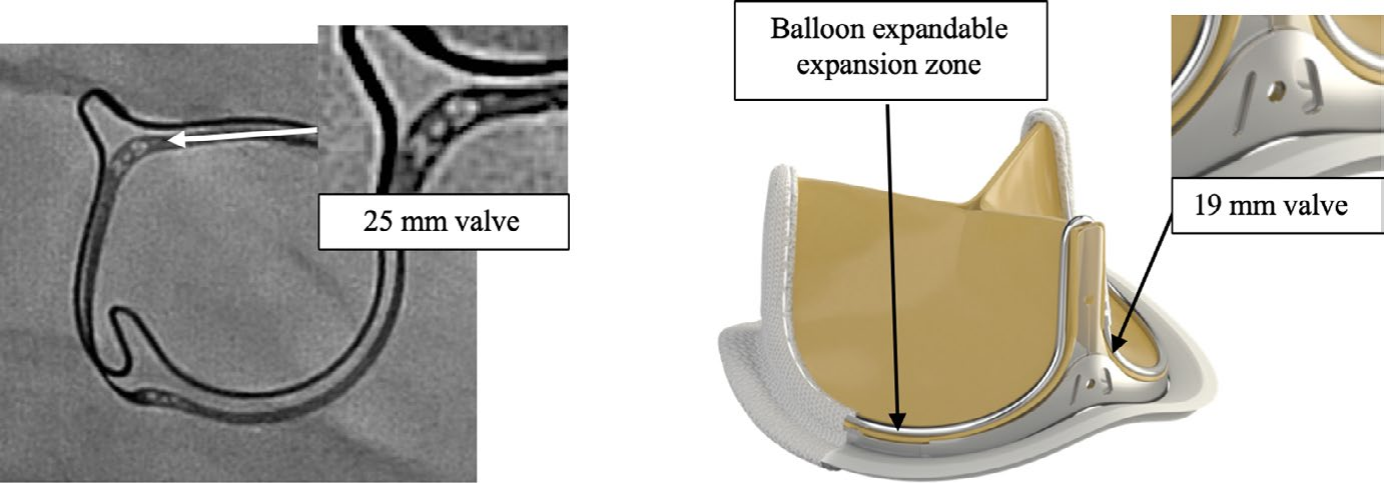
Features of the Inspiris Resilia valve that provide a valve‐in‐valve advantage. A, Radio‐opaque size identification of 25 mm Inspiris valve. B, Ex vivo Inspiris Resilia aortic valve demonstrating the radiographic marker (19 mm) and expansion zone

This valve has two features that optimize future valve‐in‐valve transcatheter insertion. This is especially important in adult congenital patients, where most will require repeat interventions throughout their life. The first feature is the radio‐opaque size identification visible under fluoroscopy (Figure 6A), allowing lifelong identification of the valve size for subsequent TPVR. The second feature is an expansion zone in the annulus of the prosthesis, which is also visible under fluoroscopy and can allow expansion of the valve by several millimeters during subsequent valve placement (Figure 6B). This will allow for TPVR to be done using the same or larger size and can help decrease the incidence of patient prosthetic mismatch.^9^

These features and our short‐term experience have demonstrated the Inspiris Resilia valve is a promising prosthesis for the adult congenital population. It has the potential to provide longer periods free of reintervention and may optimize for future transcatheter valve procedures. Our experience prompts further inquiry to evaluate its long‐term efficacy and durability in the pulmonary position.

## CONFLICT OF INTEREST

None declared.

## AUTHOR CONTRIBUTIONS

CCD‐N: extracted patient history and vital imaging data from the medical records, drafted manuscript, assisted in completing edits, and submitted the manuscript. MFK: extracted patient history and vital imaging data from the medical records, drafted manuscript, and assisted in completing edits. RMS; JS; NM; VM; CIM; and AM: helped with the conceptual design of the manuscript and provided oversight to all aspects of the manuscript and contributed with his expertise and skill in the field.
